# Professor Jameson

**Published:** 1835

**Authors:** 


### IX.—Professor Jameson.

This gentleman, in accepting the chair of *Surgery*, in the Cincinnati College, has transferred his family to this city, for permanent residence. Those citizens of the west and south, therefore, who may wish to avail themselves of the ripe experience of this eminent surgeon, will have an opportunity of visiting him in Cincinnati; and such physicians as desire to purchase his Medical Journal, in two volumes, may find it in our book-stores.

